# Cutaneous manifestations of COVID-19 patients in a Hospital in São Paulo, Brazil, and global literature review^☆^

**DOI:** 10.1016/j.abd.2022.09.007

**Published:** 2023-03-10

**Authors:** Silmara da Costa Pereira Cestari, Marcela da Costa Pereira Cestari, Gabriela Franco Marques, Ivana Lirio, Reinaldo Tovo, Ilana Cruz Silva Labriola

**Affiliations:** a Hospital Sírio-Libanês, São Paulo, SP, Brasil; b Centro Universitário São Camilo, São Paulo, SP, Brasil

**Keywords:** Coronavirus infection, Pandemics, SARS-CoV-2, Skin manifestation

## Abstract

**Background:**

Since the beginning of the COVID-19 pandemic, a myriad of cutaneous manifestations have been described in association with this viral infection. However, in Latin America, this kind of data is still scarce.

**Objective:**

In this sense, the goal of this study was to describe the dermatological findings observed during SARS-CoV-2 infection, in a Brazilian Hospital.

**Methods:**

This is a cross-sectional, retrospective and descriptive study of 50 cases of new-onset dermatologic symptoms in patients with COVID-19, treated at Hospital Sírio-Libanês, from February to June 2020.

**Results:**

The patients (n = 50) were classified into 6 groups, according to the elementary lesions and the statistical analysis was performed. The most common cutaneous lesions were maculopapular eruptions (44%), necrosis, purpura, and livedo (32%), urticarial lesions (12%), pseudochilblains (4%) and papular-vesicular eruption (4%). In 46% of the patients the cutaneous lesions occurred in association with other symptoms, such as pruritus (38%), pain and burning sensation (8%). Lower limbs were affected in 44% of the cases, followed by the trunk (38%), upper limbs (24%) and face (14%). Cutaneous lesions were mostly found after other COVID-19 systemic symptoms, with a mean period between the viral syndrome and cutaneous signs of 5 days (SD = 6.1 days).

**Study limitations:**

It is a small sample, in a single-center study, with patients exclusively from a private Hospital.

**Conclusions:**

Patients in Brazil have the same proportion of lesions as revealed in other studies in Europa. The compiled data is essential for a better understanding of cutaneous manifestations deemed secondary to COVID.

## Introduction

Since its origin in the city of Wuhan, Hubei province, China, in December 2019, the new beta-coronavirus has spread rapidly, reaching Brazil in February 2020. Considered a pandemic by the World Health Organization (WHO) as of December 15, 2021; 270 million confirmed cases have been reported, including more than 5 million deaths from COVID-19.^1^

Classic symptoms of coronavirus infection include cough, fever, dyspnea, anosmia, fatigue, sore throat, and abdominal pain. However, a variable spectrum of cutaneous manifestations has been described in association with the current infectious condition.^2^, ^3^, ^4^, ^5^, ^6^ A meta-analysis of prevalence studies revealed that skin lesions among COVID-19 patients were reported in four countries (China, Thailand, France, and Italy), with an overall prevalence of 1.0% among 2,621 patients.^7^ The prevalence of cutaneous manifestations among patients with COVID-19 in Europe was 6.6%, a percentage higher than the 0.2% observed in Asia.^7^ According to published case reports and case series, more than 30 types of skin lesions have been reported among patients with COVID-19.^7^ The reported findings ranged from those most commonly seen in viral infections, such as morbilliform rashes and urticaria, to more unique ones, such as chilblain-like skin lesions and varicelliform rashes.^8^, ^9^, ^10^, ^11^, ^12^, ^13^ In relation to Latin America there is limited data about COVID-19 and skin lesions, especially in Brazil there is only one previous study.^12^

The pathophysiological mechanisms involved in the occurrence of skin lesions in patients with COVID-19 remain poorly understood.^14^ It is believed that the mechanisms involved in cutaneous signs and symptoms might be similar to those found in other viral exanthemas, resulting from an immune response to viral nucleotides, which culminates in the release of large amounts of pro-inflammatory cytokines; or else, to be secondary to systemic effects caused by COVID-19, especially in cases of vasculitis and thrombotic vasculopathy.^15^, ^16^

The scope of knowledge about the proper incidence of skin lesions secondary to COVID infection, its patterns, and the possible specificity of these lesions with this virus remains to be determined in specialized literature, requiring further studies for better understanding. Moreover, the relationship between these types of presentations and prognosis is yet to be fully established. Because many gaps and doubts linger, the authors aimed to present a study that intended to describe the dermatological findings observed during SARS-CoV-2 infection in patients treated at a large private hospital in São Paulo, also assessing possible mechanisms involved in skin lesions and their relationship with the severity of the disease. In that vein, better strategies to prevent and treat possible dermatological complications in individuals with COVID-19 could be created, improving the ability to manage resources when the course of the disease is known in such scenarios and the descriptive data of these subjects become potential predictors of prognostic markers such as skin lesions.

## Material and methods

This is a cross-sectional, retrospective, and descriptive study. Data was gathered from all patients admitted to the emergency department of Sírio-Libanês Hospital (São Paulo, Brazil) presenting with COVID-19 symptoms and cutaneous manifestations, between February and June 2020. Individuals who did not require hospitalization, and showing mild to moderate COVID-19 symptoms, were evaluated by a dermatologist only during the period of care in the emergency department. Patients requiring hospitalization were followed up by physicians from the dermatology team throughout the full length of in-hospital time. Whenever consent was given, skin lesions were photographed without any identification of the specific patient. Cases were selected according to the following criteria: 1) Subjects with COVID-19 symptoms, regardless of severity, 2) A positive nose swab (PCR test) for COVID-19; 3) Presence of skin lesions of recent onset (previous 2 weeks) documented by photo, at the time of the clinical evaluation in the emergency department, or during the hospitalization period. Skin biopsy was not performed routinely, being utilized only in cases demanding additional anatomopathological examination for diagnostic elucidation, abiding by institutional norms of consent agreed by the patient. Information originated from medical records included age at disease onset, gender, clinical features of COVID-19, and relevant medical history. The cutaneous manifestations were described by dermatologists according to the patterns of skin lesions. It was also recorded the topography, and the time of onset in relation to the COVID-19 symptoms, as well as the symptoms associated with the dermatological findings. Prognostic factors of possible worse outcomes were also analyzed in hospitalized patients, such as the need for non-invasive ventilation, intubation, dialysis, Extracorporeal Membrane Oxygenation (ECMO), and death.

Microsoft Excel® was used as the data sheet template, allowing analysis for descriptive statistics. The continuous variables were presented as mean, median, minimum, and maximum values, and Standard Deviation (SD); while the categorical variables were presented as absolute (n) and relative (%) frequencies. Statistical analysis was performed using IBM SPSS Statistics Version 24 and statistical significance was set at 5% (p < 0.05). The inferential analyzes used in order to confirm or refute evidence found in the descriptive analysis were *t*-Student for independent samples, Mann-Whitney, Analysis of Variance (ANOVA) with one factor Fixed, Kruskal-Wallis, Pearson's Chi-Square and Fisher's Exact or its extension.

This study was approved by the Ethics Committee of Sírio-Libanês Hospital, under the number 32840120.4.0000.5461, and written informed consent was obtained from all the participants.

## Results

The authors collected 50 cases of new-onset dermatologic symptoms in patients with laboratory-confirmed COVID-19 treated at Sírio-Libanês Hospital from February to June 2020. The main demographic and clinical findings are summarized in Table 1. The age of the patients ranged between 18 to 99 years, with a mean of 57 years (SD = 18.6 years), and 54% were male. Most patients (74%) had some type of comorbidity and 76% were hospitalized for a mean period of 17.7 days, ranging from 0 to 120 days. Among patients with a more severe disease, 46% required non-invasive ventilation, 32% were under invasive ventilation, 8% were submitted to dialysis, 4% needed ECMO and only one patient died.

**Table 1:** Patients with COVID-related skin lesions treated at Sírio-Libanês Hospital, Sâo Paulo, Brazil.

Variables	Results (n = 50)
Male, n (%)	27 (54%)
Mean age, years ± SD	57 ± 18.6
Hospitalized patients, n (%)	38 (76%)
Average hospitalization time, days ± SD	17.7 ± 25
Non-invasive ventilation	23 (26%)
Intubation	16 (32%)
Dialysis	8 (16%)
Extracorporeal membrane oxygenation (ECMO)	2 (4%)
Death	1 (2%)
Skin lesions, n (%)	
Maculopapular eruption	22 (44%)
Ischemic phenomena (necrosis, purpura, or livedo)	16 (32%)
Urticarial rash	6 (12%)
Pseudochilblains	2 (4%)
Papular-vesicular eruption	2 (4%)
Symptoms in skin lesions, n (%)	
None	27 (54%)
Pruritus	19 (38%)
Pain and burning sensation	4 (8%)
Target skin sites, n (%)	
Lower extremities	22 (44%)
Trunk	19 (38%)
Upper extremities	12 (24%)
Face	7 (14%)
Disseminated lesions (two or more body segments)	7 (14%)
Time between initial COVID and cutaneous manifestations, days ± SD	5 ± 6.1
Systemic symptoms, n (%)	49 (98%)
Cough	31 (63.3%)
Fever	30 (61.2%)
Myalgia	30 (61.2%)
Dyspnea	25 (51%)
Headache	14 (28.6%)
Anosmia	10 (20.4%)

**Figure 1:**
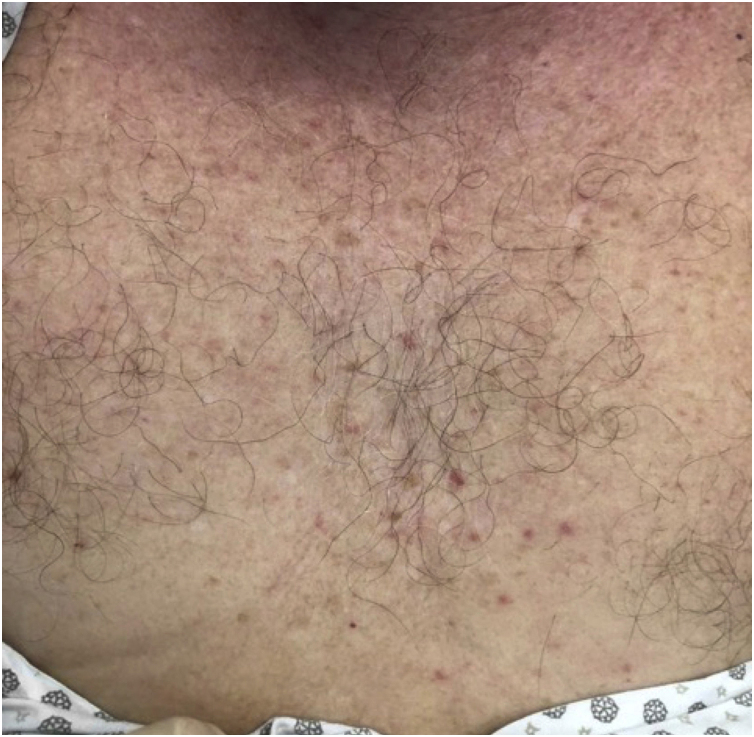
Erythematous rash containing macules and papules.

**Figure 2:**
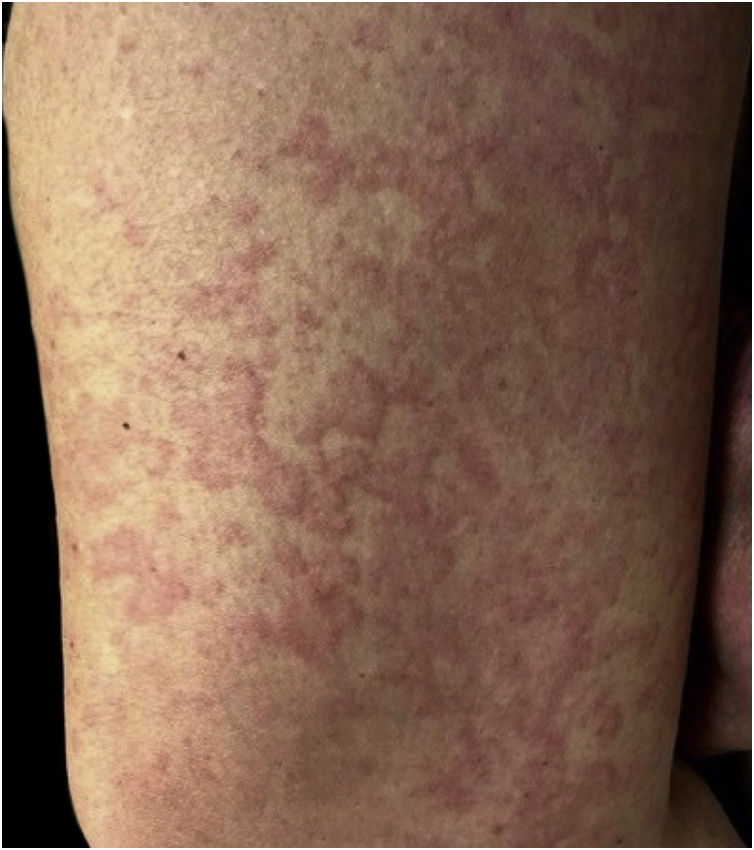
Urticarial rash.

**Figure 3:**
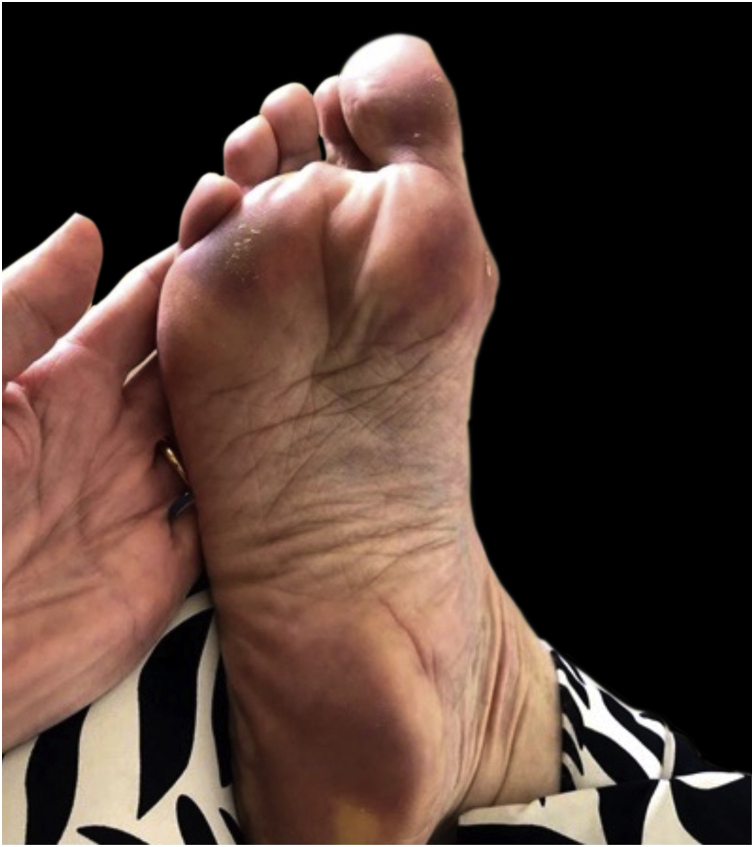
Pseudochilblains.

The majority presented maculopapular eruption (44%) (Fig. 1), followed by ischemic phenomena, necrosis, purpura, and livedo (32%), urticarial lesions (12%) (Fig. 2), pseudochilblains (4%) (Fig. 3), papulovesicular eruption (4%) and other injuries such as thrush (4%). In 46% of the patients, cutaneous lesions occurred in association with other symptoms, such as pruritus (38%), pain and burning sensation (8%). The pruritus was most common in urticarial lesions and in papulovesicular eruptions (p < 0.001). Regarding pain and burning sensation, there were no statistical differences between the categories of injuries. Patients with livedo and papulovesicular eruption had a higher intubation rate (p = 0.002), however, the other factors that suggested worse prognosis (e.g., dialysis, ECMO and death), did not show statistical differences. Younger patients were more likely to present itching (p = 0.035), with cutaneous lesions predominantly on the trunk (p < 0.001) and less commonly on the lower limbs (p = 0.049). In addition, patients who presented pruritus had less anosmia (p = 0.003).

The general distribution of skin lesions was 44% in the lower limbs, followed by the trunk (38%), upper limbs (24%) and face (14%). Specifically, the maculopapular and papulovesicular lesions were preferentially found on the trunk (p < 0.001) while the pseudochilblains and vascular lesions (livedo, ischemia, necrosis, and purpura) were preferentially found in lower limbs (p = 0.036). Patients with lesions on the face have more symptoms of burning (p = 0.017).

Cutaneous lesions were usually found after other COVID-19 symptoms, with a mean period between the viral syndrome and cutaneous signs of 5 days, ranging from 0 to 39 days. The most frequent COVID-19 symptom observed was cough (63.3%), followed by fever (61.2%), myalgia (61.2%), dyspnea (51%), headache (28.6%) and anosmia (20.4%). Although an anecdotal description of systemic symptoms and cutaneous manifestations is possible, prompting potential patterns yet to be fully proven, the present sample did not show statistical significance concerning this aspect of the disease (p = 0.560).

## Discussion

Even after two years of the beginning of the pandemic, there are still limited data available in the world literature regarding the cutaneous manifestations related to COVID-19. In many of these studies, the diagnosis of COVID-19 was based on clinical criteria, without confirmation by molecular tests. In the present study, the present sample consisted only of confirmed cases of COVID-19 after RT-PCR, and the skin lesions of all patients were evaluated by dermatologists.

Regarding the age of onset of COVID-19 patients with skin lesions, the present sample was in line with current literature, presenting a mean of 57 years of age. A recent systematic review reported a 60% prevalence of cutaneous manifestations and dermatological sequelae of COVID-19 infection in patients with a mean age of 56.9 years.^2^ According to studies, patients with COVID-19 of all age groups can present skin lesions, ranging from children under one-year-old to elderly individuals.^17^, ^18^ The prevalence according to sex has divergent findings between studies; some report a slight predominance among women,^2^ while others have observed a higher prevalence among men.^9^, ^10^, ^19^

The most common morphologies observed in a multicenter study with 171 patients with laboratory-confirmed COVID-19 from 31 countries were morbilliform rash (22%), pseudochilblains (18%), urticarial (16%), macular erythema (13%), vesicular eruption (11%), papulosquamous rash (9.9%), and retiform purpura (6.4%).^20^ In Spain, a nationwide case collection survey of images and clinical data from 375 suspected and confirmed cases of COVID-19 described five major clinical patterns: erythema with vesicles or pustules (pseudochilblain) (19%), other vesicular eruptions (9%), urticarial lesions (19%), maculopapular eruptions (47%) and livedo or necrosis (6%).^21^ Studies carried out in Italy,^19^ France^10^ and Turkey^9^ observed similar cutaneous manifestations, as well as the findings of the present study (Table 2). In other series of cases, the maculopapular eruptions were the most frequent skin manifestations.^20^, ^21^

**Table 2:** Main epidemiological and clinical data of patients with COVID-related skin lesions published in the literature and in the present series.

Variables	Cestari Brazil, 2022	Marzano et al., Italy, 2021	Galván Casas et al., Spain, 2020	Jacquin-Porretaz et al., France, 2020	Askin et al., Turkey, 2020
Nº cases	50	200	375	39	52
Male	54%	54%	NR	53.9%	58.6%
Mean age, years	57	57	NR	44	57.4
Skin lesions					
Maculopapular eruption	44%	25.7%	47%	50%	23%
Livedo/Purpura/Necrosis	32%	9.0%	6%	8.3%	15%
Urticarial rash	12%	10.2%	19%	25%	13.5%
Pseudochilblain	4%	24.6%	19%	49%	1.9%
Papular-vesicular eruption	4%	15.5%	9%	16.6%	5.8%
Symptoms in skin lesions					
None	54%	NR	NR	NR	NR
Pruritus	38%	40.5%	NR	NR	NR
Pain/Burning	8%	11%	NR	NR	NR

NR, Not Reported.

The sample showed a preponderance of these patterns of cutaneous lesions, as well as the papulovesicular eruption, preferentially on the trunk^8^, ^17^; while the pseudochilblains, livedo and other vascular lesions were mostly located on the lower extremities. These findings corroborate those of the literature.^17^, ^18^, ^20^ Although skin lesions are predominantly located on the trunk and extremities; lesions on the face, neck, mouth and armpits have also been reported.^15^ Another similar data was related to symptoms associated with skin lesions, with pruritus being the most common in urticarial lesions and in papulovesicular eruptions.^8^, ^11^, ^19^

To date, a clear relationship between the cutaneous signs and the severity, or prognosis of the patient, has not been supported. Jamshidi et al. observed that skin lesions may occur either in patients with mild (48%), moderate (32%), or severely affected coronavirus infection patients (20%), with an overall mortality rate of 4.5%.^22^ Patients with vascular lesions have the highest mortality rate (18%), whereas patients with urticaria-like lesions have the lowest mortality rate (2%).^22^ In the present study, despite the high hospitalization rate (76%) and requiring invasive support measures in many cases, the mortality rate (0.02%) was lower than that found in other series.

According to published studies, pseudochilblain was more frequently observed in younger patients, with lower rates of systemic symptoms, pulmonary infiltration, laboratory abnormalities, hospitalization, and ICU admission, characterizing a less severe disease,^11^, ^23^ whereas retiform purpura and other vascular occlusion lesions were reported in critical illness.^11^, ^17^, ^20^ In the present sample, patients with livedo and papulovesicular eruption had a higher intubation rate, however, the other factors of worse prognosis, such as dialysis, ECMO, and death did not show statistical differences.

The duration of the skin lesions observed in the cases reported in the literature range from only 20 minutes to four weeks, with an average duration of a few days. According to data found in the literature, the average interval between the first systemic symptom and the advent of skin lesions ranges between 1 and 14 days. In the present study, the authors were able to determine a mean of 5 days. It is worth noticing that in some studies skin lesions were observed 2 to 5 days before the onset of classic symptoms of COVID-19.^24^

This is the first series of cases reported in Brazil with patients evaluated in person by dermatologists. The hospital where the study took place is a large private institution, being the only one in the country with a dermatological emergency department available 24 hours, which facilitated the assessment of skin lesions at the time of their appearance in patients with laboratory-confirmed COVID-19. There is a Brazilian study using self-reported skin manifestations over the phone and the prevalence of skin lesions was 31% in 1,429 confirmed infected patients. An important caveat of such work, however, is the use of self-reported only dermatologic manifestations through an electronic survey.^12^

The main limitation of this study was the difficulty to calculate the prevalence of skin lesions in patients with COVID-19, considering the fact that the total number of patients with a confirmed diagnosis was not available at the time. Additionally, it was not possible to estimate the duration of cutaneous manifestations in the entirety of the cataloged cases since a portion of the affected individuals were evaluated only during their period of care in the emergency room.

## Conclusions

Cutaneous manifestations may be present in patients with COVID-19 regardless of the intensity of the symptoms of the viral infection. They can affect patients of any age, being slightly more frequent in men. Skin lesions described deemed secondary to COVID-19 are heterogeneous and included maculopapular eruption, urticarial rash, pseudochilblains, papulovesicular eruption, livedo, necrosis, and purpura. Any part of the body can be affected, with lesions being more frequent on the trunk and limbs. Patients with livedo and papulovesicular eruption may have a worse prognosis, although more studies are warranted to establish this relationship.

## Financial support

None declared.

## Authors' contributions

Silmara da Costa Pereira Cestari: Study concept and design; data collection, or analysis and interpretation of data; statistical analysis; writing of the manuscript or critical review of important intellectual content; data collection, analysis, and interpretation; effective participation in the research guidance; intellectual participation in the propaedeutic and/or therapeutic conduct of the studied cases; critical review of the literature; final approval of the final version of the manuscript.

Marcela da Costa Pereira Cestari: Data collection, or analysis and interpretation of data; statistical analysis; writing of the manuscript or critical review of important intellectual content; data collection, analysis, and interpretation; critical review of the literature; final approval of the final version of the manuscript.

Gabriela Franco Marques: Data collection, or analysis and interpretation of data; statistical analysis; writing of the manuscript or critical review of important intellectual content; data collection, analysis, and interpretation; critical review of the literature; final approval of the final version of the manuscript.

Ivana Lirio: Data collection, or analysis and interpretation of data; statistical analysis; writing of the manuscript or critical review of important intellectual content; data collection, analysis, and interpretation; critical review of the literature; final approval of the final version of the manuscript.

Reinaldo Tovo: Study concept and design; data collection, or analysis and interpretation of data; statistical analysis; writing of the manuscript or critical review of important intellectual content; data collection, analysis, and interpretation; effective participation in the research guidance; intellectual participation in the propaedeutic and/or therapeutic conduct of the studied cases; critical review of the literature; final approval of the final version of the manuscript.

Ilana Cruz-Silva Labriola: Study concept and design; data collection, or analysis and interpretation of data; statistical analysis; writing of the manuscript or critical review of important intellectual content; data collection, analysis, and interpretation; effective participation in the research guidance; intellectual participation in the propaedeutic and/or therapeutic conduct of the studied cases; critical review of the literature; final approval of the final version of the manuscript.

## Conflicts of interest

None declared.
